# Do Nanoparticle Physico-Chemical Properties and Developmental Exposure Window Influence Nano ZnO Embryotoxicity in *Xenopus laevis*?

**DOI:** 10.3390/ijerph120808828

**Published:** 2015-07-28

**Authors:** Patrizia Bonfanti, Elisa Moschini, Melissa Saibene, Renato Bacchetta, Leonardo Rettighieri, Lorenzo Calabri, Anita Colombo, Paride Mantecca

**Affiliations:** 1 Department Earth and Environmental Sciences, POLARIS Research Centre, University of Milano Bicocca, 1 Piazza della Scienza, 20126 Milan, Italy; E-Mails: patrizia.bonfanti@unimib.it (P.B.); elisa.moschini@unimib.it (E.M.); m.saibene2@campus.unimib.it (M.S.); anita.colombo@unimib.it (A.C.); 2 Department of Biosciences, University of Milan, 26 via Celoria, 20133 Milan, Italy; E-Mail: renato.bacchetta@unimi.it; 3 Tec Star S.r.l., Viale Europa, 40, 41011 Campogalliano, Italy; E-Mails: rettighieri@tec-star.it (L.R.), calabri@tec-star.it (L.C.)

**Keywords:** zinc oxide, nanoparticles, *Xenopus laevis*, FETAX, surface coating, nanotoxicology

## Abstract

The growing global production of zinc oxide nanoparticles (ZnONPs) suggests a realistic increase in the environmental exposure to such a nanomaterial, making the knowledge of its biological reactivity and its safe-by-design synthesis mandatory. In this study, the embryotoxicity of ZnONPs (1–100 mg/L) specifically synthesized for industrial purposes with different sizes, shapes (round, rod) and surface coatings (PEG, PVP) was tested using the frog embryo teratogenesis assay-*Xenopus* (FETAX) to identify potential target tissues and the most sensitive developmental stages. The ZnONPs did not cause embryolethality, but induced a high incidence of malformations, in particular misfolded gut and abdominal edema. Smaller, round NPs were more effective than the bigger, rod ones, and PEGylation determined a reduction in embryotoxicity. Ingestion appeared to be the most relevant exposure route. Only the embryos exposed from the stomodeum opening showed anatomical and histological lesions to the intestine, mainly referable to a swelling of paracellular spaces among enterocytes. In conclusion, ZnONPs differing in shape and surface coating displayed similar toxicity in *X. laevis* embryos and shared the same target organ. Nevertheless, we cannot exclude that the physico-chemical characteristics may influence the severity of such effects. Further research efforts are mandatory to ensure the synthesis of safer nano-ZnO-containing products.

## 1. Introduction

The explosion of the nanotech revolution implies that new and previously-unknown materials are introduced into the environment, generating new ecological relationships among living and non-living systems, with unpredictable scenarios for the long-term effects on human and environmental health. 

Trying to fill the gap between the use of nanomaterials (NMs) and the possible health risks, the newborn nanotoxicology discipline has the mission to unravel the toxicological properties of the huge number of NMs already employed and to orient safe nanotech future development.

In the vast NM catalogue, nano-metal oxides (nMeOs) represent one of the more widely-used categories in industrial applications, and they are globally produced in thousands of tons per year. 

After nanosized titanium dioxide (nTiO_2_), nano-zinc oxide (nZnO) was the most abundantly produced [1]. Its action as a stabilizer agent has promoted the use in food, cosmetics and other consumer products, such as paints, and recently, it has attracted great interest for its UV-protective and antibacterial capacities, which make it suitable for a wide range of applications [2]. 

Accordingly, nZnO represents one of the prioritized NM to be considered for regulation as confirmed by the abundant literature available about its toxicological effects. 

Many studies investigated nZnO effects on human cells and laboratory mammals, pointing out the relevant cytotoxic and inflammatory potency of this NM [3]. Besides, nZnO probably represents the only NM that has been uncontrovertibly associated with a specific human disease, metal fume fever a chronic inflammatory status manifested in workers chronically-exposed to welding fume [4]. 

Adverse effects after nZnO exposure were reported also in aquatic organisms throughout the trophic chain [5,6]; nevertheless, it has been used as a dietary supplement in human and livestock [7]. Several papers agree about attributing to nZnO a heavy acute toxic effect on different ecologically-relevant groups, like algae, bacteria and crustaceans [8,9,10,11]. With respect to vertebrates, nZnO was seen to be adversely affecting zebrafish embryos and adults [12,13,14,15], as well as the normal development of the amphibian *Xenopus laevis* [16,17,18]. In two previous papers, we demonstrated that nZnO specifically targets gut development, producing histological and molecular effects as a function of NP dimension, the smaller NPs being the most effective [16,19]. 

Since the nanotoxicity studies targeting the reproductive and developmental aspects are rather scanty and considering that the nZnO mechanism of action during embryogenesis is not fully understood, in this work, we investigated the relationships between the ZnONP properties and the developmental alterations produced. 

In particular, we focused attention on the comparative embryotoxic effects of differently-shaped and -coated ZnONPs obtained from a supplier, who developed different formulations of nano-ZnO to be used as antibacterial and UV filter fillers for polymers and paints.

Smaller, round *vs.* bigger, rod NPs and PVP and PEG surface-coated *vs.* uncoated NPs were tested in order to establish which NP properties might be involved more than others in inducing the specific toxicity outputs and, thus, possibly listed to be considered as targets in a safe-by-design study. In addition, we performed further assays by exposing embryos throughout different developmental windows to characterize which embryonic stages are more sensitive to nZnO exposure. Our findings mainly show that the different ZnONPs induce similar embryotoxic effects, targeting the same organ, the intestine, with ingestion as the primary uptake route. The surface coating with PEG seems a possible way to reduce the embryotoxicity of ZnONPs during *Xenopus* development.

## 2. Materials and Methods

### 2.1. Chemicals and NPs Used

All analytical-grade reagents, human chorionic gonadotropin (HCG), 3-amino-benzoic acid ethyl ester (MS222), salts for FETAX solution and ZnSO_4_ were purchased from Sigma-Aldrich S.r.l., Italy.

The different ZnONPs used were supplied by TecStar S.r.l. (Campogalliano, Modena, Italy); they were produced by gas phase pyrolysis methods. 

The ZnONPs used are indicated as follow: sZnO (smaller, round NPs), bZnO (bigger, rod). These NPs tested here are both nude and surface-coated with polyvinylpyrrolidone (PVP10K) or polyethylene glycol (PEG400) and indicated as PVP-sZnO, PEG-sZnO, and so on.

The functionalization of nanoparticles is obtained by TecStar proprietary wet chemical procedures.

All suspensions and stock solutions were prepared in FETAX, whose composition in mg/L was 625 NaCl, 96 NaHCO_3_, 30 KCl, 15 CaCl_2_, 60 CaSO_4_·2H_2_O and 70 MgSO_4_, at pH 7.6–8.0. Test suspensions (1, 10, 50 and 100 mg/L) were sonicated for 10 min in a Branson 2510 sonifier and stored in the dark at 4 °C.

### 2.2. NP Characterization

The size and morphology of uncoated NPs were investigated by high-resolution scanning electron microscopy (HR-SEM) equipped with a field emission electron source (FEI STRATA DB235M, 30-kV beam voltage). ZnONPs (sZnO, bZnO) were also characterized by transmission electron microscopy (TEM). For this purpose, they were suspended in distilled water, sonicated for 1 min and vortexed. Aliquots of 3 µL of NP suspension (100 mg/L) were immediately pipetted and deposited onto Formvar®-coated 200 mesh copper grids; the excess of water was gently blotted by filter paper. Once dried, grids were directly inserted into a Jeol-JEM1220 transmission electron microscope operating at 100 kV, and images were taken using a dedicated Lheritier LH72WA-TEM camera.

The crystalline size and phase of uncoated NPs were investigated by X-ray diffraction (XRD) analysis (Panalytical XPERT PRO with Cu anode). 

### 2.3. Characterization of NP Suspensions

Dynamic light scattering (DLS) and inductively coupled plasma optical emission spectrometry (ICP-OES) measurements were performed to characterize the NP hydrodynamic behavior and dissolution, respectively, in FETAX medium. 

For DLS and Z-potential measurement, a Nanosizer ZS (Malvern Instruments Ltd) was used. Suspensions of coated and uncoated sZnO and bZnO were prepared in FETAX solution and immediately analyzed; the reported values represent the mean of five independent measures.

To estimate the NP dissolution and the possible contribution to the toxicity of Zn^2+^ dissolved in FETAX medium during the exposure, the suspensions of coated and uncoated bZnO and sZnO were collected after 24 h and 96 h from the beginning of the test. 

Then, they were ultrafiltrated using centrifuge tubes VIVASPIN 6 with a molecular weight cut-off of 10,000 Da (Sartorius Stedim Biotech GmbH, Goettingen, Germany). The Zn^2+^ concentration in ZnONP-free ultrafiltrated solutions was measured by ICP-OES with a Perkin-Elmer Optima 7000 DV (Perkin-Elmer, Santa Clara, CA, USA). The analyses were conducted on samples from two independent bioassays, and each measurement was replicated three times. 

### 2.4. FETAX Assay

Adult *X. laevis* were purchased from Centre de Ressources Biologiques *Xénopes* (Université de Rennes 1, Rennes Cedex), maintained in aquariums with dechlorinated tap water at a 22 ± 2 °C, alternating 12-h light/dark cycles and fed a semi-synthetic diet (Mucedola S.r.L., Settimo Milanese, Italy) three times a week.

The FETAX test was run according to the standard protocol ASTM [20]. Embryos were obtained from the natural breeding of pairs of adult *X. laevis* previously injected with HCG in the dorsal lymph sac (females: 300 IU; males: 150 IU). Breeding tanks were filled with FETAX solution and well aerated before introducing the couples. Amplexus normally ensued within 2–6 h, and the deposition of fertilized eggs occurred from 9–12 h after injection. After breeding, adults were removed and embryos collected and degelled with 2.25% of L-cysteine in FETAX solution (pH 8.0). Normally-cleaved embryos at the midblastula stage (Stage 8), 5 h post-fertilization (hpf) [21], were selected for testing and placed in 6.0-cm glass Petri dishes containing 10 mL of control or test solution. For each female, the plates were duplicated. All of the Petri dishes were incubated in a thermostatic chamber at 23 ± 0.5 °C until the end of the test (96 hpf), and each day, the test solutions were renewed and the dead embryos removed. At this moment, mortality and malformation data were generated as endpoints of the assay. For each experimental group, the number of dead larvae was recorded, and survivors were anaesthetized with MS-222 at 100 mg/L and evaluated for single malformations by examining each specimen under a dissecting microscope. At the end of the bioassays, surviving normal larvae were formalin fixed for growth retardation measurements. Each assay was repeated at least three times under the same experimental conditions.

### 2.5. Experimental Design

The experimental design was set up as follows: (1) to probe the embryotoxic potency of differently-sized and -shaped ZnONPs, a conventional FETAX assay (exposure over Stages 8–46) was conducted by exposing embryos to sZnO and bZnO at increasing concentrations of 1, 10, 50 and 100 mg/L; (2) to test a possible influence of surface coating in the observed embryotoxic properties, further comparative FETAX assays (Stages 8–46) were performed using PVP- and PEG-coated sZnO and bZnO at the effective concentration of 50 mg/L; (3) to establish the NP uptake route and the most sensitive developmental windows, further experiments were conducted by exposing embryos to sZnO and bZnO from Stage 8 (mid-blastula, 5 hpf; beginning of FETAX assay) to Stage 39 (2 days 8 hpf; opening of the stomodeum), corresponding to the likelihood for NP ingestion, and from Stage 39–Stage 46 (4 days 10 hpf; end of the primary organogenesis that is the end of the FETAX assay); the results obtained from these different exposure conditions were compared to the results from the exposure during the whole embryogenetic period (Stages 8–46); (4) at the end of the test (96 hpf), pools of Stage 46 embryos of each exposure condition and exposed to an effective concentration of 50 mg/L were randomly selected and immediately stored at −80 °C for measurement of superoxide dismutase (SOD) enzymatic activity, a biomarker of oxidative stress, or processed for light and electron microscopy analyses.

### 2.6. Superoxide Dismutase Enzymatic Activity 

Total SOD activity (Cu/Zn-, Mn- and Fe-SOD) was quantified by the SOD Assay Kit (Cayman, Ann Arbor, MI, USA) according to the manufacturer’s instructions. The test uses a tetrazolium salt to detect superoxide radicals generated by xanthine oxidase. One unit (U) of SOD activity corresponds to the quantity of enzyme yielding 50% dismutation of superoxide radical.

Pools of 20 embryos, collected from each treatment group exposed to the effective concentration of 50 mg/L, were homogenized in 1 mL of 20 mM cold HEPES buffer (pH 7.2). Then, the homogenates were centrifuged at 1500× *g* for 5 min at 4 °C. A volume of 200 μL of radical detection solution was added to 10 μL of the supernatants or SOD standard solutions in a 96-well plate. The reaction was initiated by adding xanthine oxidase solution, and absorbance was measured at 450 nm with a multiplate reader (Multiskan Ascent Thermo Scientific Co., Italy). Data were normalized for the protein content of each sample, determined by the BCA method using BSA as a standard, and expressed as mean specific SOD activity (U/mg proteins) ± SEM of three independent experiments.

### 2.7. Light and Electron Microscopy Analyses

For light and TEM analyses, embryos were randomly selected at the end of the FETAX assays and fixed in 2.5% glutaraldehyde in 0.1 M sodium cacodylate buffered solution at pH 7.4. After several washes in the same buffer, larvae were post-fixed in 1% OsO_4_ for 1.5 h at 4 °C, dehydrated in a graded ethanol series, then transferred in 100% propylene oxide. Infiltration was subsequently performed with propylene oxide and embedding resin (Araldite-Epon) at volumetric proportions of 2:1 for 1.5 h, 1:1 overnight and, finally, 1:2 for 1.5 h. Embryos were then left in 100% pure resin for 4 h, and polymerization was performed at 60 °C for 48 h. Semi-thin sections of 0.5 μm were obtained by a Reichert Ultracut E microtome, collected onto microscope slides and stained with 1% toluidine blue to be screened under the light microscope and to select the region of interest for TEM observations. Ultra-thin sections of 50 nm of the intestinal loops were collected on 200-mesh uncoated copper grids and not counterstained to avoid contaminations by lead citrate and uranyl acetate that ultimately may interfere with metal NP visualization. Samples were analyzed using a Jeol JEM1220 transmission electron microscope operating at an accelerating voltage of 80 kV and equipped with a Lheritier LH72WA-TEM digital camera.

### 2.8. Data Collection and Statistical Analysis

The number of dead embryos *versus* their total number at the beginning of the test led to the mortality percentages, and the number of malformed larvae *versus* the total number of surviving ones gave the malformed larva percentages. Data are presented as the average ± SEM. The data were tested for homogeneity and normality. When these assumptions were met, one-way analysis of variance (ANOVA) was performed, and otherwise, the non-parametric Kruskal–Wallis test was applied. The significance level was set at *p* < 0.05. The incidence of specific malformations was investigated with chi-square, with Yates’s correction for continuity (χ2 test) or Fisher’s exact tests (FE test). Concentrations causing 50% lethality or malformation at 96 hpf were calculated, when possible, and classified as lethal (LC_50_) or teratogenic (TC_50_), respectively. These were obtained following the elaboration of the lethality and malformation data by the probit analysis [22], using the U.S. EPA Probit Analysis Program, Version 1.5. The Teratogenic Index (TI), useful in estimating the teratogenic risk associated with the tested compounds, is represented by the LC_50_/TC_50_ ratio [23]. 

## 3. Results

### 3.1. NP Physical and Chemical Characteristics

**Figure 1:**
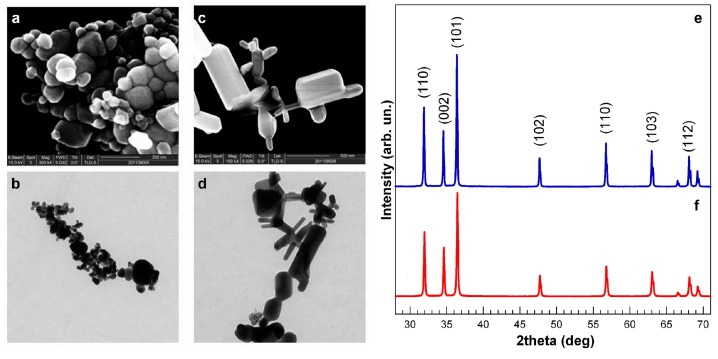
Physical and chemical characterization of ZnO nanoparticles. SEM and TEM images of sZnO (smaller, round) (**a**,**b**) and bZnO (bigger, rod) (**c**,**d**). XRD analysis of dry sZnO (**e**) and bZnO (**f**); the main planes for zincite crystal are reported.

SEM and TEM pictures clearly show that the sZnO sample (Figure 1a,b) is made of round NPs, while bZnO (Figure 1c,d) is composed of bigger, rod-shaped NPs. The mean sizes for sZnO and bZnO (maximum dimension) were 63 ± 29 nm and 334 ± 208 nm, respectively.

The XRD pattern of dry ZnO NPs was studied using a diffraction angle 28°–71°. All of the peaks have 100% phase matching with the ZnO hexagonal phase of zincite crystal, and no other characteristic impurity peaks were detected (Figure 1e,f).

The line broadening in the peaks determines the crystallite size of ZnO, and the average crystalline size of dry ZnO NPs can be estimated by the well-known Scherrer relation.

Table S1 reports the main results obtained from DLS analysis of hydrodynamic diameters and surface charge (Z-potential) and XRD analysis of crystalline size.

The use of mechanical and ultrasonication techniques was not enough to obtain homogeneous suspensions of particles in FETAX medium. All of the ZnONPs show the tendency to aggregate, as testified by the values of the hydrodynamic diameter determined by DLS.

Nevertheless, coated ZnONPs seemed to be separated from each other by dispersion techniques in a more efficient way; this effect may be caused by steric repulsion induced by the presence of the polymeric coating on the particle surface. 

Zeta potential measurements show that sZnO has a positive charge, and the functionalization of these particles with PEG and PVP slightly increases the Z-potential values. Nude and PVP-coated bZnO show a negative charge, while PEG-bZnO a positive one. Anyway values of the Z-potential in the range of −30 mV and +30 mV generally indicate unstable suspensions.

**Figure S1:**
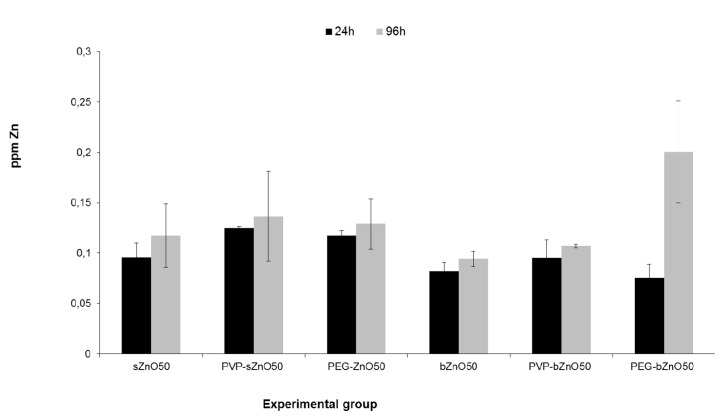
Levels of dissolved ions from sZnO and bZnO after 24 h and 96 h from the beginning of the FETAX assay measured by ICP-OES.

In our experimental conditions, the concentration of Zn^2+^ measured after 24 h and 96 h by ICP-OES was lower than 0.3 mg/L and independent of the NP incubation time for both nude and coated NPs (see Figure S1).

These results confirm that ZnO NPs are very poorly soluble in FETAX medium. 

### 3.2. Comparative Embryotoxicity of Differently-Sized and -Shaped ZnONPs

**Figure 2:**
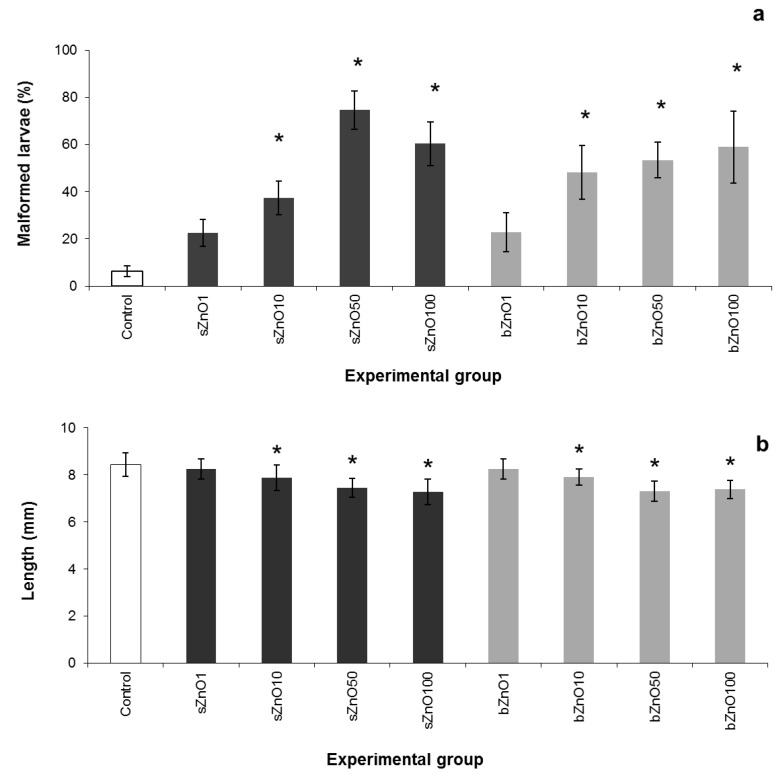
Comparative FETAX results after exposure of embryos to 1–100 mg/L of sZnO and bZnO. (**a**) Malformation rates; (**b**) growth retardation. Dark grey = sZnO-exposed larvae; light grey = bZnO-exposed larvae; bars = SEM; * statistically different from the control (*p* < 0.05, ANOVA + Fisher LSD method).

The physicochemical properties, such as size and shape, along with the effective concentration of ZnONPs were evaluated by the comparative toxicity of sZnO and bZnO on *Xenopus laevis* embryos (Figure 2). 

At the end of the test, mortality values were lower than 3% and not significantly different from the control for both NPs (data not shown). On the other hand we observed a concentration-dependent increase in malformation rates in the range of 1–50 mg/L that is statistically different from the control starting from a concentration of 10 mg/L for both sZnO and bZnO. 

The embryotoxicity of sZnO appears to be higher than that of bZnO, especially in embryos exposed to 50 mg/L, even if the malformation percentage mean values of the two types of ZnONPs were not statistically different. Nevertheless, the 96 h TC_50_ values calculated by the probit method in the range 1–50 mg/L were 17.9 mg/L for sZnO and 59.47 mg/L for bZnO, suggesting that sZnO has a higher embryotoxic potential than bZnO. However, it is not possible to calculate the TI, because of the low mortality recorded, which did not allow estimating the LC_50_.

**Figure 3:**
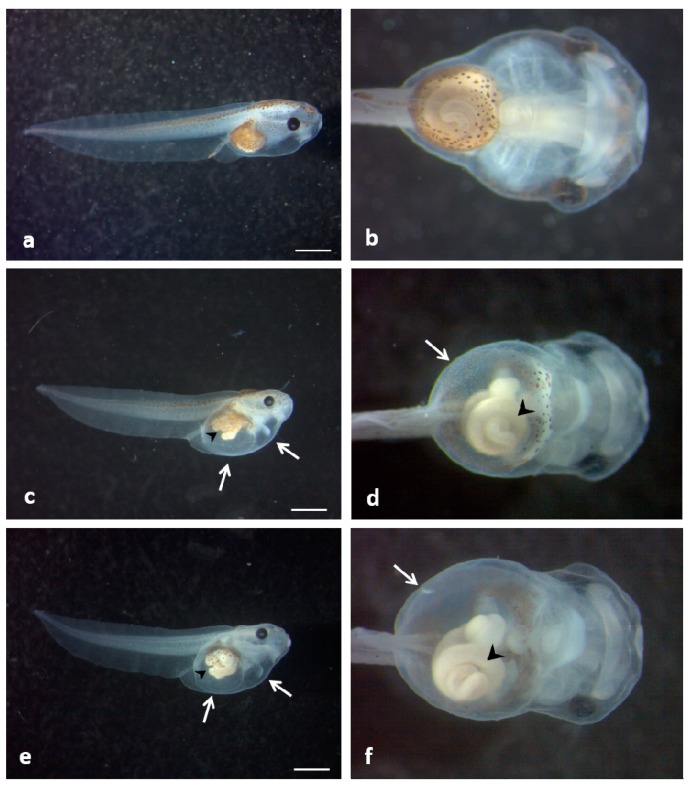
*Xenopus laevis* larvae at the end of the FETAX test. (**a**) Lateral and (**b**) ventral view of a control; (**c**) lateral and (**d**) ventral view of an embryo exposed to 50 mg /L sZnO; (**e**) lateral and (**f**) ventral view of an embryo exposed to 50 mg/L bZnO. The treated larvae show abnormal gut coiling (arrow head), abdominal and cardiac edemas (empty arrow) and a slight dorsal tail flexure. (b,d,f) Original magnification: 4×. Bars = 1 mm.

After exposure to 50 mg/L of ZnONPs, more than 70% of the embryos for sZnO and more than 50% for bZnO were abnormal; irregular gut coiling and abdominal or cardiac edema were the most frequent abnormalities observed (Figure 3).

It is noteworthy that the sZnO affected the gut coiling more heavily in comparison to bZnO, as demonstrated by the chi-square test of the specific malformations (Table 1).

As reported for the malformation rate, a significant growth retardation was observed starting from 10 mg/L for both ZnO NPs, and a concentration-dependent response was also detected up to 100 mg/L (Figure 2b). 

In conclusion, considering the comparative embryotoxicity results, sZnO and the bZnO have resulted in a significant malformation incidence and growth inhibition, in which the size and the shape of the NPs play a role.

### 3.3. Influence of Polymer Surface Coating on ZnONP Embryotoxicity

**Figure 4:**
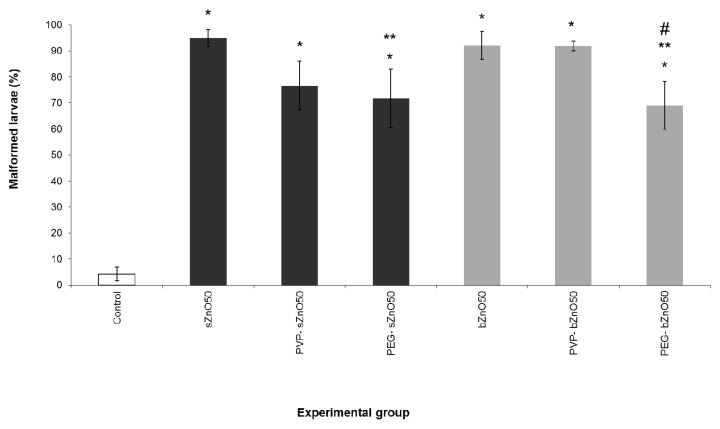
Comparative FETAX malformation percentages after exposure of embryos to nude and polymer-coated sZnO and bZnO at 50 mg/L. Dark grey = sZnO-exposed larvae; light grey = bZnO-exposed larvae. Bars = SEM; * statistically different from the control at *p* < 0.001; ** statistically different from the corresponding nude nanoparticles at *p* < 0.05; ^#^ statistically different from the corresponding PVP-coated bZnO at *p* < 0.05,ANOVA + Fisher LSD method.

Based on previous embryotoxicity experiments, 50 mg/L of ZnONPs was selected as the effective concentration in order to assess the influence of surface coating on the embryotoxicity of the considered nanoparticles. We performed a specific FETAX assay comparing nude and polymer-coated (PVP and PEG) sZnO and bZnO, and the results are shown in Figure 4.

**Table 1:** Malformation patterns in embryos exposed to sZnO and bZnO.

	sZno (mg/L)					bZnO (mg/L)			
	**Control**	**1**	**10**	**50**	**100**	**1**	**10**	**50**	**100**
Living Larvae	247	247	249	258	173	243	249	257	169
Malformation									
Severe n (%)	3 (1.2)	5 (2.0)	8 (3.2)	4 (1.6)	7 (4.0)	1 (0.4)	3 (1.2)	9 (3.5)	2 (1.2)
Gut n (%)	4 (1.6)	35 (14.2) ^b^	66 (26.5) ^b^	116 (45.0) ^b,c^	79 (45.7) ^b,c^	29 (11.9) ^b^	71 (28.5) ^b^	70 (27.2) ^b^	48 (28.4) ^b^
Edema n(%) Cardiac	0	0	2 (0.8)	31 (12.0) ^b^	4 (2.3) ^a^	2 (0.8)	32 (12.9) ^b^	12 (4.7) ^b^	0
Abdominal	8 (3.2)	15 (6.1)	34 (13.7) ^b^	90 (34.9) ^b^	39 (22.5) ^b^	19 (7.8) ^a^	70 (28.1) ^b^	57 (22.2) ^b^	47 (27.8) ^b^
Dorsal Flexure n (%)	0	0	2 (0.8)	13 (5.0) ^b^	1 (0.6)	0	0	8 (3.1) ^b^	16 (9.5) ^b^

Percentages based on the number of malformations/the number of those living. ^a^ Chi-square test; *p*<0.05 *versus* control. ^b^ Chi-square test; *p* <0.001 *versus* control. ^c^ Chi-square test; *p* <0.001 sZnO *versus* the corresponding concentration of bZnO.

No embryolethality was observed (data not shown), while it was confirmed that a high and similar incidence of malformations was induced by both sZnO and bZnO. From the comparison of the coated to the nude ZnONPs, a significant reduction in malformation rate emerged in embryos treated with PEG-coated sZnO with respect to the nude ones and in those treated with PEG-coated bZnO compared to the corresponding nude and PVP-coated nanoparticles. Similarly to what was observed in embryos treated with nude NPs, coated ZnONPs once again affected mainly the gut coiling, abdominal and cardiac cavities, causing edema. While being lower in percentage in the embryos treated with polymer-coated nanoparticles, these kinds of gross malformations were still high and statistically significant compared to the control.

These findings suggest that PEG is able to significantly reduce the damage induced by the ZnONPs, even if the embryotoxic effect remains high.

### 3.4. Ingestion-Dependent Toxicity of ZnONPs

Since the exposure during whole embryogenesis (Stages 8–46) highlighted that gut was the main target of the ZnONP embryotoxicity, two specific developmental windows were chosen to evaluate if the ingestion of ZnONPs could be responsible for the detected malformations. The first group of embryos was exposed to ZnONPs before the stomodeum opening (from Stage 8–39); in this period, the embryo surface is the only route of exposure. A second group of embryos was exposed to ZnONPs after stomodeum opening (from Stage 39–Stage 46); during these stages, embryos begin to ingest water and suspended materials. 

**Figure 5:**
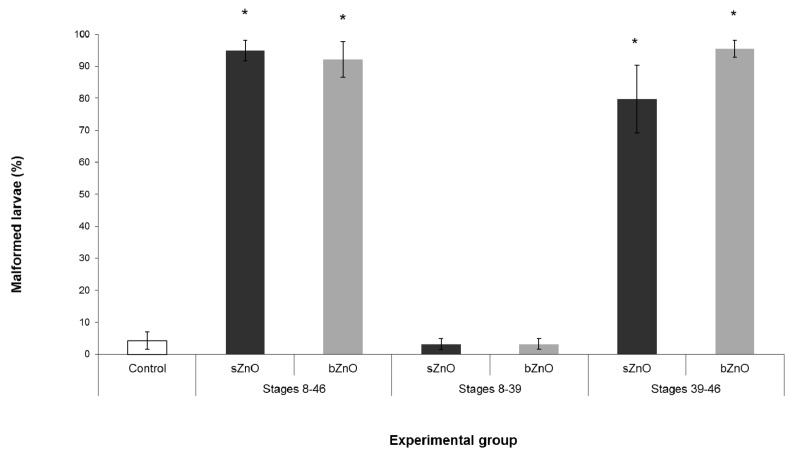
Percentages of malformed embryos after exposure to sZnO and bZnO at 50 mg/L in different developmental windows. Dark grey = sZnO-exposed larvae; light grey = bZnO-exposed larvae. Stages 8–46, exposure during the whole embryogenesis as in the FETAX protocol; Stages 8–39, exposure from blastula to the stomodeum opening (two days 8 hpf); Stages 39–46, exposure from stomodeum opening to the end of the primary organogenesis. * Statistically different from control at *p* < 0.001, ANOVA + Fisher LSD method.

Figure 5 summarize the results obtained by these different exposure conditions. 

We observed that both sZnO and bZnO at 50 mg/L induced malformation rates comparable to those of conventional FETAX only when the exposure began after stomodeum opening. On the contrary, malformation rates comparable to the control were recorded in embryo groups exposed during the first developmental window. These data suggest that ingestion represents the main route of uptake for both ZnONPs. 

### 3.5. Oxidative Stress Responses

**Figure 6:**
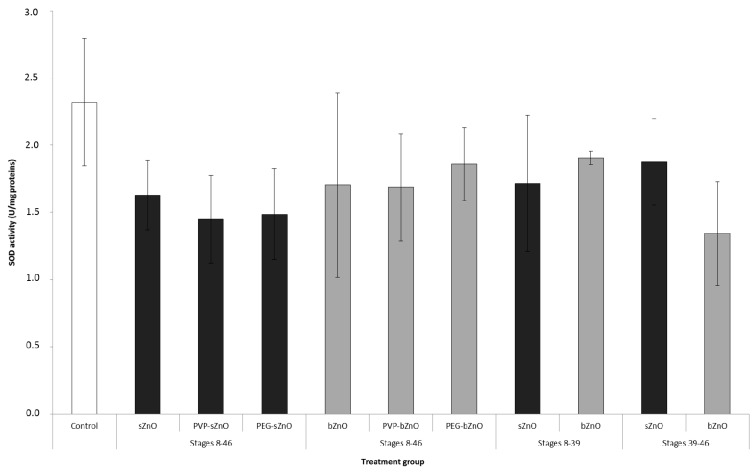
SOD enzymatic activity in embryos exposed to nude and polymer-coated sZnO and bZnO 50 mg/L during different developmental stages. Dark grey = sZnO-exposed larvae; light grey = bZnO-exposed larvae.

Oxidative stress induced by 50 mg/L of nude and polymer-coated sZnO and bZnO at Stage 46 whole embryos of all experimental groups was investigated by measuring SOD activity, which provides the first defense against ROS toxicity. As shown in Figure 6, we observed a slight, but not significant, decrease in SOD activity if compared to the control in all of the experimental groups. This result suggests that the production of ROS potentially induced by ZnONPs exposure, if any, is not able to elicit a clear alteration in SOD activity, at least if it is measured in pools of whole embryos and not in single embryos or in target organ. 

### 3.5. Histological and Ultrastructural Effects of ZnONPs on Small Intestine

**Figure 7:**
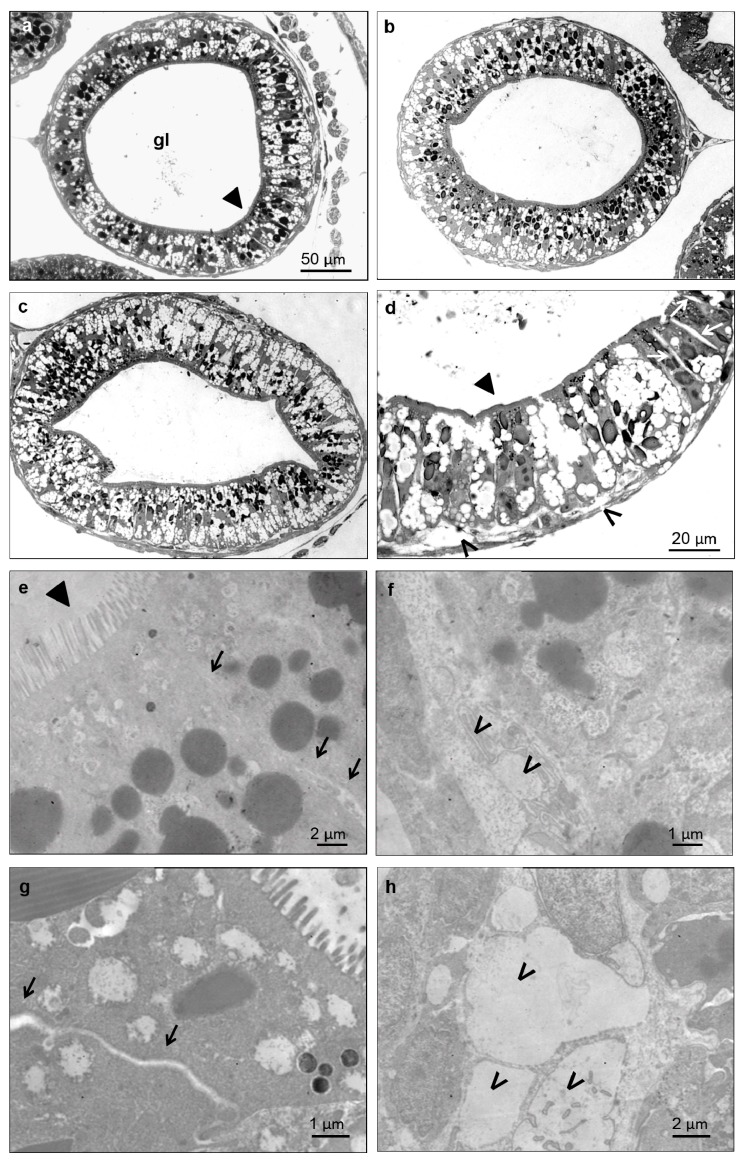
Light (**a**–**d**) and electron microscopy (**e**–**h**) imaging of the *X. laevis* small intestine. Transversal sections at the level of an intestinal loop of a control (a), bZnO (b) and sZnO (c,d) exposed embryos. Magnification of the sZnO intestinal loop (d) shows the swelling of paracellular spaces between cells (empty arrow) and detachment in some regions of epithelial cells from basal lamina (*). These damages are more evident in the detail of the junctional complex between two enterocytes (g) (black arrow) and of the basal portion (h) (*) of sZnO-exposed embryos in comparison to the control (e) (black arrow) and (f) (*). ► = brush border; gl = gut lumen.

Since abnormal gut coiling was the main feature of ZnONP-treated embryos, preliminary histological and ultrastructural analyses of small intestine were performed (Figure 7). 

Despite the severity of the gut anatomical abnormality induced by ZnONPs, no obvious signs of histological damages were noted in bZnO-treated embryos (Figure 7b), while very mild tissue lesions were observed in embryos exposed to sZnO (Figure 7c,d,g,h). These alterations mainly consisted of a swelling of paracellular spaces in intestinal mucosa and detachment of some enterocytes from the basal lamina. On the contrary, the brush border of enterocytes was not affected. 

## 4. Discussion

Thousands of papers fill the literature of the last 15 years with the toxic effects of many different nanomaterials on *in vitro* and *in vivo* systems. Nevertheless, many aspects in nanotoxicology are still critical and need substantial improvements to make this discipline mature. According to the authors, an in depth mechanistic knowledge of the NP toxicity and an increase in the efforts devoted to the study of the reproductive and developmental toxicity of new NMs should be considered mandatory in the actual second life of nanotoxicology. 

This work is our contribution to increasing the knowledge of these aspects, and it is focused on the developmental effects and the mode of action of the massively-produced and widely-used nano-zinc oxide. A panel of six ZnONPs, differing in size and shape (big and rod or small and round) and in surface coating (PEG, PVP), was used to understand if and how these NMs affect *Xenopus laevis* embryos by considering the most sensible developmental stages and the main NP target organs.

The following discussion is organized into three paragraphs according to the three different aspects evaluated.

### 4.1. Comparative Toxicity of Differently-Sized and -Shaped ZnONPs

Based on our results, sZnO and bZnO induced comparable effects on *X. laevis* embryos. No mortality was observed after exposure to both NPs, while the percentage of malformed larvae and the growth retardation significantly increased starting from the concentration of 10 mg/L (Figure 2). Anyway, sZnO was more effective at 50 mg/L, where the highest malformation score was observed, while it decreased at 100 mg/L. This result suggests a reduced bioavailability of sZnO, likely dependent on the stronger NP agglomeration at the highest concentration, as also proposed by Bai *et al.* [13] for nZnO of 30 nm in water suspension. For this reason, the calculation of TC_50_ in the tested range of 1–100 mg/L was possible only for bZnO, and it was 48.9 mg/L. Instead, TC_50_ re-calculation in the range of 1–50 mg/L was 17.9 mg/L for sZnO and 59.47 mg/L for bZnO. Taken together, these results reinforce the evidence that smaller and round-shaped ZnONPs are more embryotoxic than bigger, rod-shaped ones. Similar findings were obtained exposing *Daphnia magna* to commercial forms of ZnONPs, as reported in Santo *et al.* [11]. In this paper, the acute toxicity of small-sized particles was higher than the bigger ones. Very similar effects were also reported in our previous study on *Xenopus laevis*, where the embryotoxicity of two differently-sized commercial nZnOs similar in shape was compared [19]. 

Although it was not possible to calculate the teratogenic index, based on our results, the estimated TI should be many times greater than three, and then, sZnO and bZnO should be considered “highly teratogenic” compounds according to Dawson and Bantle [23].

In our previous papers [16,19], we already suggested the potential teratogenic action of nZnO; the news from this work is that the teratogenic effect is almost independent of the NP size and shape, although the NP physico-chemical characteristics may contribute to aggravating such an effect. 

Several papers report the toxicity of different metal oxide nanoparticles on zebrafish embryos, and many of them investigated the toxicity of nZnO [12,13,24,25,26,27]. By comparing our results to those available on zebrafish, we can argue that the sensitivity to nZnO of the amphibian and fish developing embryos should be considered quite similar. Bai and collaborators [13] observed that ZnONPs killed zebrafish embryos at 50 and 100 mg/L, while at lower concentrations, they reduced body length, induced malformations and retarded embryo hatching. Furthermore, Zhu and collaborators [15] evidenced that nZnO affects the hatching rate of zebrafish and reported an 84-h EC_50_ value of 23.06 mg/L.

Many researchers agree that embryotoxic effects are dependent on the MeONP properties rather than on the dissolved ions [13,15,24]. According to these authors, the metal cations dissolved from the NPs only partially contributed to the nZnO toxicity. On the contrary, other authors have reported that the nZnO toxicity in both *in vitro* and *in vivo* systems are strongly dependent on NP dissolution [12]. Although the question is still debated, the solubility of nZnO can be highly dependent on the suspension medium (e.g., media added with serum albumin or ions in comparison to pure water), the initial particle size and pH [28]. 

As already observed [16,19], the ZnONP dissolution in FETAX medium, a saline solution with a pH around 8.0, is very poor, and also in this study, the maximum Zn^2+^ concentration measured in NP suspension ultrafiltrates was lower than 0.5 ppm (Figure S1). No embryotoxic effects were observed in *Xenopus* embryos exposed to zinc ions from ZnSO_4_ at concentrations similar to those measured by ICP-OES according to Bacchetta *et al.* [16] and Mantecca *et al.* [29].

Based on these findings, we can affirm that in our experimental conditions, size and shape did not significantly affect NP dissolution in FETAX medium, making the contribution of Zn ions to toxicity on *Xenopus* embryos very low. 

On the contrary, although the effects elicited by the sZnO and bZnO NPs could be considered qualitatively similar, the smaller, round NPs were more effective. 

The embryotoxicity induced by 50 mg/L sZnO was slightly higher than that induced by bZnO, and sZnO affected more heavily the gut coiling in comparison to bZnO, as reported in the Results Section. No influence of size nor shape was detected on growth retardation.

These finding could be attributed to the higher surface reactivity and the easier cell uptake of sZnO. It is in fact well proven that round-shaped NPs in the size range of 10–30 nm are preferentially taken up by cells through endocytosis [30,31]. 

Looking at the results of the oxidative stress biomarker SOD (Figure 6), it is evident that nZnO exposure induced an enzymatic activity depletion, although not statistically different from the control. Again, the sZnO seemed to be more effective than bZnO. Many literature data support the oxidative changes in cells and developing embryos as the main responses to nZnO [14,26], and we also recently demonstrated that *Xenopus* embryo exposure to nZnOs results in antioxidant genes’ upregulation [19], although the correspondent increment in the enzymatic activity is not always evident. As previously discussed, this may be attributable to the limitation of having to perform the analysis on pools of whole embryos and not on a single target organ due to the small dimensions of the embryos. Moreover, the developing *Xenopus* have good antioxidant defenses, including enzymes, such as SOD and the GSH-related system [32], able to buffer the ROS production in cells during embryonic development.

### 4.2. Effects of Polymer Surface Coating 

Polymer surface coating is a technique widely used in industry to obtain final commercial products (e.g., paintings, additives) with better performances. In fact, this modification basically can improve the dispersion of poorly-soluble NPs by modifying the surface properties of particles. Several papers underline the key role of the surface properties of ZnONPs in controlling cytotoxicity, demonstrating the reduction of toxicity in *in vitro* and *in vivo* systems after exposure to coated NPs [25,33]. The specific surface area and/or surface reactivity of ZnONPs govern NP-biological interactions by regulating cellular nanoparticle uptake or altering both the intracellular or extracellular Zn dissolution. 

In our work, although the observed malformation rates after exposure to coated NPs were significantly higher compared to the control group, the results highlighted that surface modification of particles with PVP and PEG is able to decrease the embryotoxicity of ZnONPs. In particular, PEGylation appears to be more effective at reducing the toxicity of both NPs. 

Sometimes, the surface coating can improve the dispersion of poorly-soluble NPs by modifying particle aggregation and settling.

Comparing DLS results of PEG-bZnO and bZnO, we could assume that, in our experimental conditions, these particles have a quite similar hydrodynamic behavior (analogous hydrodynamic diameter, −30 mV < Z-potential < +30 mV); therefore, we can also hypothesize that the modality of larvae-NP interaction, and the amount of ingested NPs are quite comparable; nevertheless, the rate of malformed larvae is lower in the PEG-bZnO- than in the bZnO-exposed larvae. 

For the small, round-shaped ZnONPs, we can affirm that the hydrodynamic diameter of the PEG-sZnO is significantly lower than that of the sZnO. Different scenarios could be thus hypothesized. If the PEG-NPs are more stable in suspension, they are more bioavailable for larvae by swallowing from the water column, while non-PEGylated NPs are more prone to settle down, being bioavailable by larvae grazing from the bottom. In this first scenario, the decreasing in embryotoxicity after exposure to the PEG-sZnO can be explained only by a reduced toxicity of the PEGylated NPs. In a second scenario, where grazing can be considered as a major feeding behavior determining NP uptake, the reduced embryotoxicity after exposure to PEG-sZnO could be the consequence of the reduced bioavailability of the PEG-sZnO, which is more stable in suspension with respect to the uncoated ones.

In both scenarios, PEGylation appears to be effective at reducing the embryotoxicity of sZnONPs.

This is in agreement with Luo and collaborators study [33], who evidenced that the PEGylation of ZnONPs decreases their cytotoxicity in comparison to other surface modifications by reducing the cellular uptake. 

### 4.3. Route of Exposure, nZnOs Target Organs and the Sensitive Developmental Window

In our previous studies [16,19], we have already demonstrated that ZnONPs are highly embryotoxic and that gut is the main affected organ. In particular, the smallest ZnONPs tested were more effective at inducing more severe histopathological effects at the gut mucosa level, with the epithelium severely eroded [19]. In the present work, the intestine was again the target organ, and the abnormal gut coiling was the principal malformation recorded. Nevertheless, embryo histopathological screening and gut ultrastructural analysis revealed only a slight alteration of intestinal mucosa, ascribable to detachment between adjacent cells and from basal lamina, as previously described, mainly after exposure to sZnO.

The choice of performing the exposure of embryos to ZnONPs in two developmental windows (before and after stomodeum opening) allows us to demonstrate that *Xenopus laevis* embryos become more susceptible to nZnO also with the acquisition of grazing behavior following the stomodeum opening. By this route, an increasing amount of suspended and aggregated NP sedimented on the bottom of the Petri dish reach the gut lumen. Conversely, if the exposure is limited to the developmental period in which the embryo is enveloped by the fertilization membrane, the ZnONPs are no longer able to induce embryotoxicity. These results suggest that the fertilization membrane could represent a barrier toward ZnONPs or, if not, the skin of the embryos is not the preferential route for NP internalization. On the contrary, zebrafish embryos are highly sensitive to ZnONPs during early developmental stages due to the high solubility of zinc in zebrafish culture medium and because of the NP interaction with chorion, affecting hatching [13,24]. 

## 5. Conclusions

ZnONPs differing in size, shape and polymeric surface coating produced significant and qualitatively similar toxicity in *X. laevis* embryos.

Nevertheless, this work points out that the ingestion is the main exposure route, and the gut is the most sensitive organ in developing *Xenopus* embryos exposed to nZnO. Specific physical and chemical characteristics affect the mode of action of these NPs and influence the severity of the effects. Smaller, round ZnONPs were more effective than bigger, rod-shaped ZnONPs, while PEGylation seemed to be effective at reducing the toxicity of the sZnONPs. 

From one side, our results evidence the potential adverse effects of nZnO on environmental health; from the other side, they suggest the possibility of playing with NP properties during the synthesis in order to modulate the toxic effects and to produce safer NMs.

## Supplementary

**Table S1:** Characterization by DLS of ZnONPs in FETAX medium and XRD analysis.

	Hydrodinamic Diameter (nm)	Zeta Potential (mV)	Average Crystalline Size (nm)
sZnO	819	+3.7	70
bZno	579	−12.8	98
PVP-sZnO	355	+15.3	-
PEG-sZnO	237	+19.6	-
PVP-bZnO	454	−10.5	-
PEG-bZnO	685	+3.8	-

## References

[1] Bondarenko O., Juganson K., Ivask A., Kasemets K., Mortimer M., Kahru A. (2013). Toxicity of Ag, CuO and ZnO nanoparticles to selected environmentally relevant test organisms and mammalian cells *in vitro*: A critical review. Arch. Toxicol..

[2] Nohynek G.J., Antignac E., Re T., Toutain H. (2010). Safety assessment of personal care products/cosmetics and their ingredients. Toxicol. Appl. Pharmacol..

[3] Pandurangan M., Kim D. (2015). *In vitro* toxicity of zinc oxide nanoparticles: A review. J. Nanopart. Res..

[4] Gerberding J.L. (2005). Toxicological Profile for Zinc.

[5] Skjolding L.M., Winther-Nielsen M., Baun A. (2014). Trophic transfer of differently functionalized zinc oxide nanoparticles from crustaceans (*Daphnia magna*) to zebrafish (*Danio rerio*). Aquat. Toxicol..

[6] Ates M., Arslan Z., Demir V., Daniels J., Farah I.O. (2015). Accumulation and toxicity of CuO and ZnO nanoparticles through waterborne and dietary exposure of goldfish (*Carassius auratus*). Environ. Toxicol..

[7] Rincker M.J., Hill G.M., Link J.E., Meyer A.M., Rowntree J.E. (2005). Effects of dietary zinc and iron supplementation on mineral excretion, body composition, and mineral status of nursery pigs. J. Anim. Sci..

[8] Aruoja V., Dubourguier H.C., Kasemets K., Kahru A. (2009). Toxicity of nanoparticles of CuO, ZnO and TiO_2_ to microalgae *Pseudokirchneriella subcapitata*. Sci. Total Environ..

[9] Blinova I., Ivask A., Heinlaan M., Mortimer M., Kahru A. (2010). Ecotoxicity of nanoparticles of CuO and ZnO in natural water. Environ. Pollut..

[10] Heinlaan M., Ivask A., Blinova I., Dubourguier H.C., Kahru A. (2008). Toxicity of nanosized and bulk ZnO, CuO and TiO_2_ to bacteria Vibrio fischeri and crustaceans *Daphnia magna* and *Thamnocephalus platyurus*. Chemosphere.

[11] Santo N., Fascio U., Torres F., Guazzoni N., Tremolada P., Bettinetti R., Mantecca P., Bacchetta R. (2014). Toxic effects and ultrastructural damages to *Daphnia magna* of two differently sized ZnO nanoparticles: Does size matter?. Water Res..

[12] Brun N.R., Lenz M., Wehrli B., Fent K. (2014). Comparative effects of zinc oxide nanoparticles and dissolved zinc on zebrafish embryos and eleuthero-embryos: Importance of zinc ions. Sci. Total Environ..

[13] Bai W., Zhang Z., Tian W., He X., Ma Y., Zhao Y., Chai Z. (2010). Toxicity of zinc oxide nanoparticles to zebrafish embryo: A physicochemical study of toxicity mechanism. J. Nanopart. Res..

[14] Xiong D., Fang T., Yu L., Sima X., Zhu W. (2011). Effects of nano-scale TiO_2_, ZnO and their bulk counterparts on zebrafish: Acute toxicity, oxidative stress and oxidative damage. Sci. Total Environ..

[15] Zhu X., Wang J., Zhang X., Chang Y., Chen Y. (2009). The impact of ZnO nanoparticle aggregates on the embryonic development of zebrafish (*Danio rerio*). Nanotechnology.

[16] Bacchetta R., Santo N., Fascio U., Moschini E., Freddi S., Chirico G., Camatini M., Mantecca P. (2012). Nano-sized CuO, TiO_2_ and ZnO affect *Xenopus*
*laevis* development. Nanotoxicology.

[17] Nations S., Wages M., Cañas J.E., Maul J., Theodorakis C., Cobb G.P. (2011). Acute effects of Fe_2_O_3_, TiO_2_, ZnO and CuO nanomaterials on *Xenopus*
*laevis*. Chemosphere.

[18] Nations S., Long M., Wages M., Canas J., Maul J.D., Theodorakis C., Cobb G.P. (2011). Effects of ZnO nanomaterials on *Xenopus*
*laevis* growth and development. Ecotoxicol. Environ. Saf..

[19] Bacchetta R., Moschini E., Santo N., Fascio U., Del Giacco L., Freddi S., Camatini M., Mantecca P. (2014). Evidence and uptake routes for Zinc oxide nanoparticles through the gastrointestinal barrier in *Xenopus*
*laevis*. Nanotoxicology.

[20] American Society for Testing and Materials (1998). Standard Guide for Conducting the Frog Embryo Teratogenesis Assay-Xenopus (FETAX).

[21] Nieuwkoop P.D., Faber J. (1956). Normal Table of *Xenopus*
*Laevis* (Daudin).

[22] Finney D.J. (1971). Probit Analysis.

[23] Dawson D.A., Bantle J.A. (1987). Development of a reconstituted water medium and preliminary validation of the frog embryo teratogenesis assay—*Xenopus* (FETAX). J. Appl. Toxicol..

[24] Zhu X., Zhu L., Duan Z., Qi R., Li Y., Lang Y. (2008). Comparative toxicity of several metal oxide nanoparticle aqueous suspensions to Zebrafish (Danio rerio) early developmental stage. J. Environ. Sci. Health Tox. Hazard. Subst. Environ. Eng..

[25] Xia T., Zhao Y., Sager T., George S., Pokhrel S., Li N., Schoenfeld D., Meng H., Lin S., Wang X., Wang M., Ji Z., Zink J.I., Madler L., Castranova V., Nel A.E. (2011). Decreased dissolution of ZnO by iron doping yields nanoparticles with reduced toxicity in the rodent lung and zebrafish embryos. ACS Nano.

[26] Zhao X., Wang S., Wu Y., You H., Lv L. (2013). Acute ZnO nanoparticles exposure induces developmental toxicity, oxidative stress and DNA damage in embryo-larval zebrafish. Aquat. Toxicol..

[27] Chen T.-H., Lin C.-C., Meng P.-J. (2014). Zinc oxide nanoparticles alter hatching and larval locomotor activity in zebrafish (*Danio rerio*). J. Hazard. Mater..

[28] Reed R.B., Ladner D.A., Higgins C.P., Westerhoff P., Ranville J.F. (2012). Solubility of nano-zinc oxide in environmentally and biologically important matrices. Environ. Toxicol. Chem..

[29] Mantecca P., Moschini E., Bonfanti P., Fascio U., Perelshtein I., Lipovsky A., Chirico G., Bacchetta R., Del Giacco L., Colombo A., Gedanken A. (2015). Toxicity evaluation of a new Zn-doped Cuo nanocomposite with highly effective antibacterial properties. Toxicol. Sci..

[30] Soenen S.J.H., Himmelreich U., Nuytten N., De Cuyper M. (2011). Cytotoxic effects of iron oxide nanoparticles and implications for safety in cell labelling. Biomaterials.

[31] Albanese A., Tang P.S., Chan W.C. (2012). The effect of nanoparticle size, shape, and surface chemistry on biological systems. Annu. Rev. Biomed. Eng..

[32] Rizzo A., Li Y., Kudera S., Della Sala F., Zanella M., Parak W.J., Cingolani R., Manna L., Gigli G. (2007). Blue light emitting diodes based on fluorescent CdSe/ZnS nanocrystals. Appl. Phys. Lett..

[33] Luo M., Shen C., Feltis B.N., Martin L.L., Hughes A.E., Wright P.F., Turney T.W. (2014). Reducing ZnO nanoparticle cytotoxicity by surface modification. Nanoscale.

